# Genomic characterization of carbapenem-resistant *Acinetobacter baumannii* from a specialized orthopaedic hospital in Southeast China, with phenotypic analysis of biofilm formation

**DOI:** 10.3389/fcimb.2026.1904047

**Published:** 2026-07-24

**Authors:** Tengfei Shi, Shaohan Xu, Xuexin Zheng, Weiqiang Shi, Rui Zhang, Hong Chen, Tengyi Chen, Huiyu Chen

**Affiliations:** 1 Department of Clinical Laboratory, Fuzhou Second General Hospital, Fuzhou, Fujian, China; 2 The Graduate School of Fujian Medical University, Fuzhou, Fujian, China; 3 Department of Clinical Laboratory, Fuzhou Second General Maternity And Child Health Care Hospital, Fuzhou, Fujian, China; 4 Department of Clinical Laboratory, Fujian Maternity and Child Health Hospital College of Clinical Medicine for Obstetrics and Gynaecology and Paediatrics, Fujian Medical University, Fuzhou, Fujian, China

**Keywords:** biofilm, carbapenem- resistant *Acinetobacter baumannii*, KL3, orthopaedic hospital, ST2, whole-genome sequencing

## Abstract

**Background:**

Carbapenem−resistant *Acinetobacter baumannii* (CRAB) is a major threat to hospitalised patients, particularly in intensive care units and orthopaedic wards where implant−associated infections are common. However, the genomic and biofilm characteristics of CRAB in orthopaedic specialty hospitals remain poorly understood.

**Methods:**

A total of 97 non-duplicate CRAB isolates collected between 2024 and 2025 were included. Antimicrobial susceptibility testing was performed using the VITEK-2 system, and biofilm formation was quantitatively assessed by the crystal violet method. Whole-genome sequencing (WGS) was carried out on the Illumina platform to analyse multilocus sequence typing (MLST), capsular types, resistance genes and virulence genes. A phylogenetic tree was constructed based on single-nucleotide polymorphisms (SNPs).

**Results:**

ST2 was the dominant clone (95.88%) among the 97 isolates, and KL3 was the most prevalent capsular type (83.51%). All isolates carried intrinsic *blaOXA-51-like* genes, predominantly *blaOXA-66* (95.88%). The most common acquired carbapenemase gene was *blaOXA-23* (98.97%), and two isolates carried metallo-β-lactamase (MBL) genes (*blaNDM-1* and *blaNDM-5*, respectively). All isolates exhibited a multidrug-resistant phenotype, with low resistance rates to tigecycline (6.19%) and minocycline (7.22%), and all remained susceptible to colistin. Strong biofilm formers accounted for 91.75% of isolates, and the carriage rates of biofilm-associated genes (*bap*, *csuABCDE*, *pgaABCD*) exceeded 90%. Phylogenetic analysis grouped the isolates into three clonal clades, with the majority (88.66%) falling into Clade C (ST2/KL3). This clade had been circulating in China as an outbreak lineage since 2018, gradually replacing Clade B (ST2/KL2), and became the dominant clone in 2024–2025.

**Conclusion:**

CRAB isolates in this orthopaedic specialty hospital are dominated by the ST2/KL3 clone, which carries multiple resistance and virulence genes, exhibits a remarkably strong biofilm-forming ability, and shows a capsular switch trend from KL2 to KL3. Enhanced molecular surveillance of this dominant clone and increased attention to anti-biofilm strategies for orthopaedic implant-related infections are strongly recommended.

## 1 Introduction

*Acinetobacter baumannii* (*A. baumannii*) is a non-fermentative, aerobic, Gram-negative bacillus widely found in healthcare environments. It readily colonises the skin, respiratory tract, gastrointestinal tract and genitourinary tract of hospitalised patients and frequently causes severe healthcare-associated infections in immunocompromised individuals (Brek et al., 2025; Cui et al., 2026; Wu et al., 2026). The organism has a remarkable ability to acquire antimicrobial resistance. Since the late 1990s, carbapenem-resistant *A. baumannii* (CRAB) has gradually emerged and spread globally (Allewijn et al., 2026). According to data from the China Antimicrobial Resistance Surveillance System (CHINET), the carbapenem resistance rate of *A. baumannii* has risen from approximately 30-40% in 2005 to over 70% in recent years (Hu et al., 2022; Yao et al., 2024). In 2017, the World Health Organization (WHO) listed CRAB as one of the most critical pathogens and placed it in the highest priority category for the development of new antibiotics (Jesudason, 2024; Sánchez-Urtaza et al., 2025; Cui et al., 2026), highlighting the urgent need for novel therapeutic and preventive strategies.

Studies from different regions have shown that global clone 2 (GC2, corresponding to ST2 under the Pasteur multilocus sequence typing(MLST) scheme) is the predominant lineage of CRAB in China and many other parts of the world (Müller et al., 2023; Morgado et al., 2023; Duan et al., 2024; Cui et al., 2026). GC2 often exhibits a multidrug-resistant (MDR) or extensively drug-resistant (XDR) phenotype. Its main carbapenem resistance mechanism is the acquisition of the *blaOXA-23* gene. This gene encodes a class D oxacillinase. Upstream of *blaOXA-23* an ISAba1 insertion sequence is frequently present, providing a strong promoter that drives high-level expression of the gene and thereby confers high-level resistance to carbapenems (Wong et al., 2019). *blaOXA-23* is usually located within composite transposons such as Tn 2006 or Tn 2009, from which it can be transferred horizontally between strains via plasmids or the chromosome, accelerating the spread of resistance (Dong et al., 2022; Xu et al., 2024). Currently, *blaOXA-23* is the most prevalent acquired carbapenemase gene among CRAB worldwide (Wei et al., 2024). In addition, GC2 carries various other resistance genes, including β−lactamase genes (*blaADC-25*, *blaTEM-1D*), aminoglycoside−modifying enzyme genes (*aph(3″)-Ib*, *aph(3′)-Ia*, *aph(6)-Id*), tetracycline efflux pump genes (*tet(B)*) and sulphonamide resistance genes (*sul2*). The synergistic action of these genes contributes to the MDR phenotype of GC2 (Wang W. et al., 2024).

Apart from extensive drug resistance, the pathogenicity of *A. baumannii* is closely linked to several virulence factors, among which biofilm formation and capsular polysaccharides play important roles, particularly in healthcare-associated infections involving indwelling medical devices (Naseef Pathoor et al., 2024). Biofilms are crucial for infection persistence and antibiotic tolerance. Key genetic determinants of biofilm formation include the *csuABCDE* (pilus assembly) gene clade, the *bap* (biofilm-associated protein) gene, the *pgaABCD* (PNAG synthesis) gene clade and the *ompA* (outer membrane protein) gene (Ali et al., 2017; Zeighami et al., 2019; Dixit et al., 2024). Capsular polysaccharides also play a complex role in virulence and resistance. They can mask virulence surface structures of *A. baumannii*, actively modulate the host immune response and affect antimicrobial resistance (Singh et al., 2018). Mutations that impair the synthesis of sugar precursors involved in capsule and lipopolysaccharide production render the bacterium susceptible to multiple antibiotic classes (Geisinger and Isberg, 2015).

Orthopaedic patients often undergo invasive surgery and receive implants (e.g., joint prostheses, fracture fixation devices), followed by prolonged intensive care, which greatly increases the risk of device-related hospital infections (Kocur et al., 2021). A*. baumannii* has a strong ability to form biofilms; biofilms act as physical barriers that protect bacteria from host immune defences and antibiotic penetration, thereby promoting persistent colonisation and chronic infection (Naseef Pathoor et al., 2024). Post-operative wound infections, osteomyelitis and implant-associated infections caused by CRAB are extremely difficult to treat and frequently lead to prolonged hospital stays, repeated surgical debridement and even implant removal (Vasso et al., 2022; Zhang et al., 2024). Therefore, a thorough understanding of the genomic background and biofilm-forming potential of CRAB in orthopaedic hospitals is of great clinical importance. Although the epidemiology and genomic features of CRAB have been extensively studied in general tertiary hospitals, especially intensive care units (Hu et al., 2022; Brek et al., 2025; Allewijn et al., 2026; Cui et al., 2026; Wu et al., 2026), systematic genomic surveillance and characterisation of CRAB in orthopaedic specialty hospitals remain scarce.

To address this gap, we conducted a two-year collection of non-duplicate clinical CRAB isolates in a tertiary hospital with a strong orthopaedic focus (the largest orthopaedic specialty group in the region) in Southeast China. A total of 97 CRAB isolates were selected for whole-genome sequencing (WGS). The aim was to systematically analyse the molecular characteristics and biofilm-forming ability of CRAB in this setting, thereby providing a basis for developing locally tailored infection control strategies and optimising antimicrobial therapy.

## 2 Materials and methods

### 2.1 Study design

A total of 97 non-duplicate CRAB isolates were collected from clinical specimens throughout the hospital between January 2024 and December 2025. CRAB was defined as an *A. baumannii* isolate resistant to imipenem or meropenem (minimum inhibitory concentration ≥8 μg/mL). Only the first isolate from each patient during a single hospitalisation was included to avoid duplication. Each isolate was evaluated by a team of clinicians and microbiologists based on the patient’s clinical signs and symptoms, inflammatory markers, response to antimicrobial therapy, and microbiological findings, in order to exclude obvious colonizing strains. All recovered isolates were re-identified, subjected to antimicrobial susceptibility testing and biofilm formation assays, and analysed by WGS for capsular typing, MLST, virulence genes and resistance genes. A phylogenetic tree was constructed based on single-nucleotide polymorphisms (SNPs). The study was approved by the Ethics Committee of Fuzhou Second General Hospital (approval No. 2024011).

### 2.2 Strain recovery, identification and antimicrobial susceptibility testing

After recovery from −80 °C stock, isolates were double-checked for species identification using matrix-assisted laser desorption/ionisation time-of-flight mass spectrometry (MALDI-TOF MS, Bruker, Germany) and the VITEK-2 automated system (bioMérieux, France).

Antimicrobial susceptibility was determined by the VITEK-2 system with the accompanying AST-N335 card (bioMérieux, France) using broth microdilution. The following 18 antibiotics were tested: cefoperazone-sulbactam (SCF), ampicillin-sulbactam (SAM), piperacillin-tazobactam (TZP), ticarcillin-clavulanate (TCC), ceftazidime (CAZ), ceftriaxone (CRO), cefepime (FEP), amikacin (AMK), imipenem (IPM), meropenem (MEM), trimethoprim-sulfamethoxazole (SXT), tobramycin (TOB), minocycline (MNO), doxycycline (DOX), colistin (COL), levofloxacin (LVX), ciprofloxacin (CIP) and tigecycline (TGC). Breakpoints for colistin and tigecycline were based on expert consensus (Society of Clinical Microbiology and Infection of China International Exchange and Promotion Association for Medical and Healthcare et al., 2020); for cefoperazone-sulbactam, the breakpoints were interpreted with reference to those of cefoperazone. The remaining results were interpreted according to CLSI M100, 35th edition. Pseudomonas aeruginosa ATCC 27853 and Escherichia coli ATCC 25922 were used as quality control strains for species identification and susceptibility testing.

### 2.3 Whole-genome sequencing

WGS was performed by Shanghai Majorbio Bio-pharm Technology Co., Ltd. (Shanghai, China). Genomic DNA was extracted using a magnetic bead-based bacterial DNA extraction kit (Majorbio, China). After quantification with the Qubit™ dsDNA HS Assay Kit (Illumina, USA), the DNA was randomly sheared into 200–400 bp fragments using a Covaris ultrasonicator (Covaris, Inc., USA). A sequencing library was prepared with the NEXTFLEX Rapid DNA-Seq Kit (Illumina, USA), followed by adapter ligation, size selection and enrichment. Paired-end sequencing (2 × 150 bp) was then performed on an Illumina NovaSeq Xplus platform (Illumina, USA). Raw sequencing data were quality-controlled using fastp (v.0.20.0) to remove adapter sequences, low-quality reads and reads with excessive N content. Clean data were assembled *de novo* with SOAPdenovo (v2.04), and the best scaffolds were taken as the final assembly. Detailed assembly statistics are provided in **Supplementary Table 2**.

### 2.4 Capsular typing, multilocus sequence typing and gene annotation

Capsular typing (K locus) and lipooligosaccharide (OC locus) typing were performed with Kaptive (v3.2.1). MLST was carried out online via the PubMLST database (https://pubmlst.org) for “*A. baumannii*”, using both the Oxford and Pasteur schemes (Jolley et al., 2018). Virulence genes were annotated with the VFanalyzer tool of the Virulence Factor Database (http://www.mgc.ac.cn/VFs/), with the genus set to “Acinetobacter”. Resistance genes were identified using ResFinder (v4.6.0), with thresholds set at ≥90% sequence identity and ≥60% coverage of the reference gene length.

### 2.5 Phylogenetic analysis

A total of 187 CRAB genome sequences from six continents, 16 countries and 22 Chinese provinces between 2010 and 2025 were downloaded from NCBI. These sequences together with the 97 isolates from this study were uploaded to the Galaxy platform (https://usegalaxy.cn/). Using Snippy (v4.6.0), all Illumina reads were mapped to the reference genome *A. baumannii* ACICU (accession no. GCF_000018445.1). Recombination-filtered polymorphic sites were extracted with Gubbins (v2.4.1), and a final phylogenetic tree was built with IQ-TREE (v2.4.0). The tree was visualised and annotated using the online tool tvBOT (https://www.chiplot.online/tvbot.html). Pairwise SNP distances between isolates were calculated with snp-dists (v0.8.2), and a threshold of ≤15 SNPs was used to define recent clonal transmission.

### 2.6 Biofilm formation assay

Biofilm formation was measured using a crystal violet microtiter plate assay. A bacterial suspension of 0.5 McFarland standard was prepared in tryptic soy broth (TSB) and diluted 100-fold with TSB. 200 μL of the diluted suspension was added to each well of a 96-well flat-bottom polystyrene plate, with three replicate wells per strain. Blank medium served as the negative control, and *A. baumannii* ATCC 19606 served as the positive control. After static incubation at 37 °C for 24 h, the supernatant was gently removed, and each well was washed three times with 200 μL of sterile phosphate-buffered saline (PBS) to remove planktonic bacteria. The wells were then fixed with 200 μL of methanol for 20 min at room temperature. After removal of the methanol and air-drying, 200 μL of 1% (w/v) crystal violet aqueous solution was added and left at room temperature for 15 min. The dye was discarded, and the wells were washed three times with 200 μL PBS and dried for 20 min at room temperature. 200 μL of 30% (v/v) acetic acid was then added to each well, and the optical density at 570 nm (OD_570_) was measured after complete dissolution. The cut-off ODc was defined as the mean OD of the negative control plus three standard deviations. Biofilm formation was classified as: none (OD ≤ ODc), weak (ODc < OD ≤ 2 × ODc), moderate (2 × ODc < OD ≤ 4 × ODc), or strong (OD > 4 × ODc).

## 3 Results

### 3.1 Source and clinical characteristics of the isolates

Among the 97 CRAB isolates, 70.10% were from male patients, and the median age was 65.1 years (IQR 24.5 years). By department, the intensive care unit (ICU) accounted for the largest proportion (34.02%), followed by internal medicine (26.80%), surgery (20.62%) and orthopaedics (17.53%). The main specimen types were respiratory samples (sputum 35.05%, bronchoalveolar lavage fluid 27.84%), wound secretions (18.56%), urine (9.28%) and others (9.27%). Among the primary diagnoses, orthopaedic diseases and neurological diseases were the most common, each accounting for 26.8%. Although the proportion of orthopaedic patients was relatively low, the high prevalence of orthopaedic diseases and wound secretion isolates suggests that post−operative orthopaedic wound infection is an important clinical manifestation of CRAB infection. Detailed information is shown in **Table 1**.

**Table 1:** Clinical characteristics of the 97 patients with CRAB infection.

Characteristic	Patients (n = 97)
Age, median (IQR) (years)	65.1(24.5)
Male, n (%)	68 (70.10)
Primary diagnosis, n (%)	
Orthopaedic diseases	26 (26.80)
Neurological diseases	26 (26.80)
Respiratory diseases	19 (19.59)
Digestive diseases	12 (12.37)
Urinary diseases	5 (5.15)
Other systemic diseases	5 (5.15)
Infectious diseases	4 (4.12)
Department, n (%)	
ICU	33 (34.02)
Internal Medicine	26 (26.80)
Surgery	20 (20.62)
Orthopaedics	17 (17.53)
Paediatrics	1 (1.03)
Specimen type, n (%)	
Sputum	34 (35.05)
Bronchoalveolar lavage fluid	27 (27.84)
Wound secretion	18 (18.56)
Urine	9 (9.28)
Bile	3 (3.09)
Ascitic fluid	2 (2.06)
Blood	2 (2.06)
Synovial fluid	1 (1.03)
Cerebrospinal fluid	1 (1.03)

IQR, interquartile range; ICU, intensive care unit.

### 3.2 Antimicrobial susceptibility

**Figure 1:**
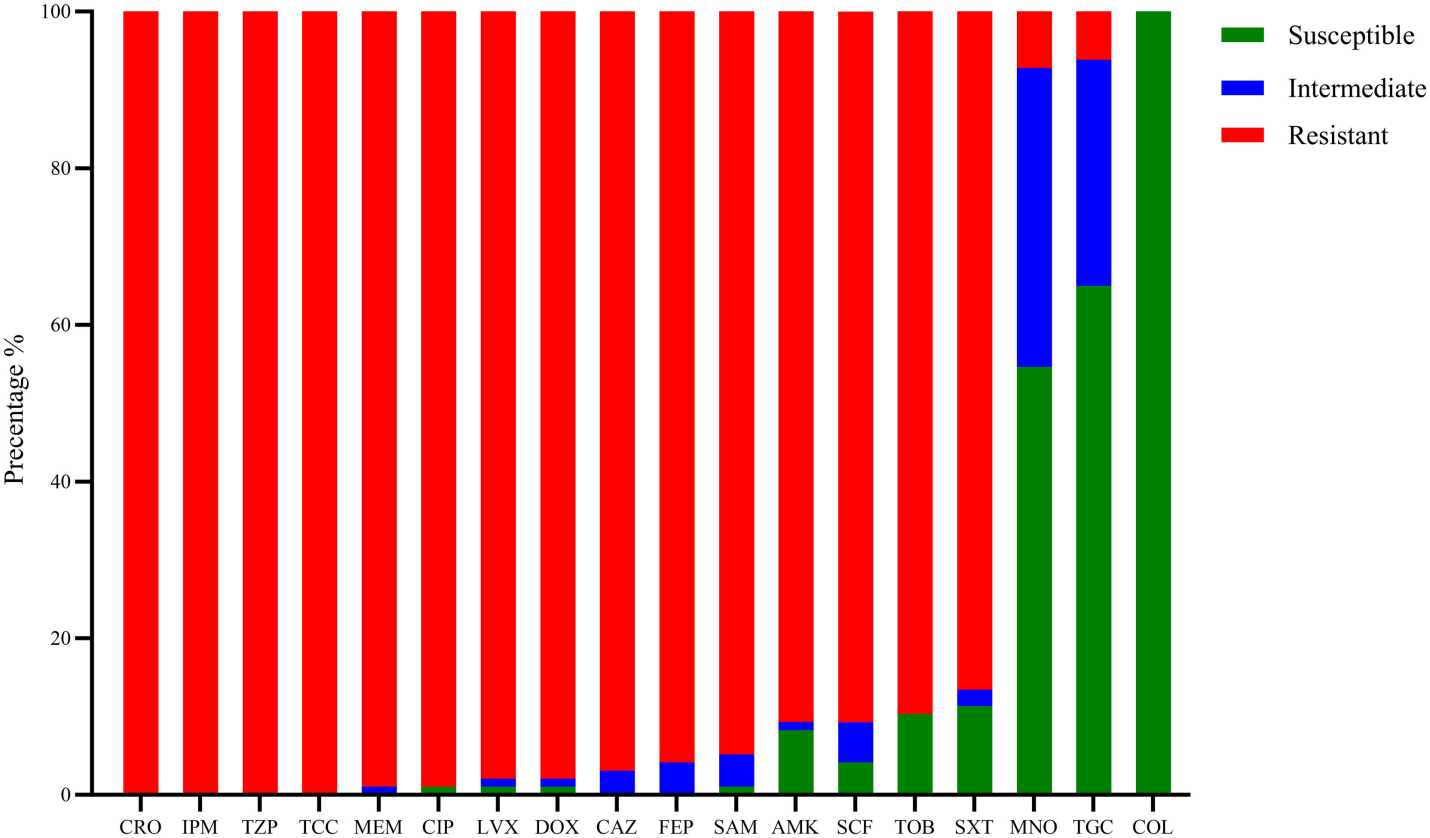
Antimicrobial susceptibility of 97 CRAB isolates. Resistance rates exceeded 95% for up to 10 antimicrobial agents, whereas lower resistance rates were observed for minocycline (7.22%) and tigecycline (6.19%). No polymyxin−resistant isolates were identified. The percentages of resistant (red bars), intermediate (blue bars) and susceptible (green bars) isolates are shown for each tested antibiotic. The following 18 antibiotics were tested: ceftriaxone (CRO), imipenem (IPM), piperacillin-tazobactam (TZP), ticarcillin-clavulanate (TCC), meropenem (MEM), ciprofloxacin (CIP), levofloxacin (LVX), doxycycline (DOX), ceftazidime (CAZ), cefepime (FEP), ampicillin-sulbactam (SAM), amikacin (AMK), cefoperazone-sulbactam (SCF), tobramycin (TOB), trimethoprim-sulfamethoxazole (SXT), minocycline (MNO), tigecycline (TGC), and colistin (COL). Data are expressed as the percentage of resistant (R), intermediate (I) and susceptible (S) isolates.

The 97 CRAB isolates showed extensive resistance to the tested antibiotics (**Figure 1**). Resistance rates were 100% for CRO, IPM, TZP and TCC; and exceeded 95% for MEM (98.97%), CIP (98.97%), LVX (97.94%), DOX (97.94%), CAZ (96.91%) and FEP (95.88%). Resistance rates to MNO and TGC were low (7.22% and 6.19%, respectively). All isolates were susceptible to COL. The most common resistance pattern was observed in 60 isolates (61.9%), which were resistant to 15 antibiotics (SAM, CRO, AMK, IPM, LVX, MEM, SXT, TOB, FEP, CAZ, TZP, TCC, DOX, SCF, CIP). For additional details, see **Table S1** in the **Supplementary Material**.

### 3.3 Multilocus sequence typing and clonal relatedness

**Figure 2:**
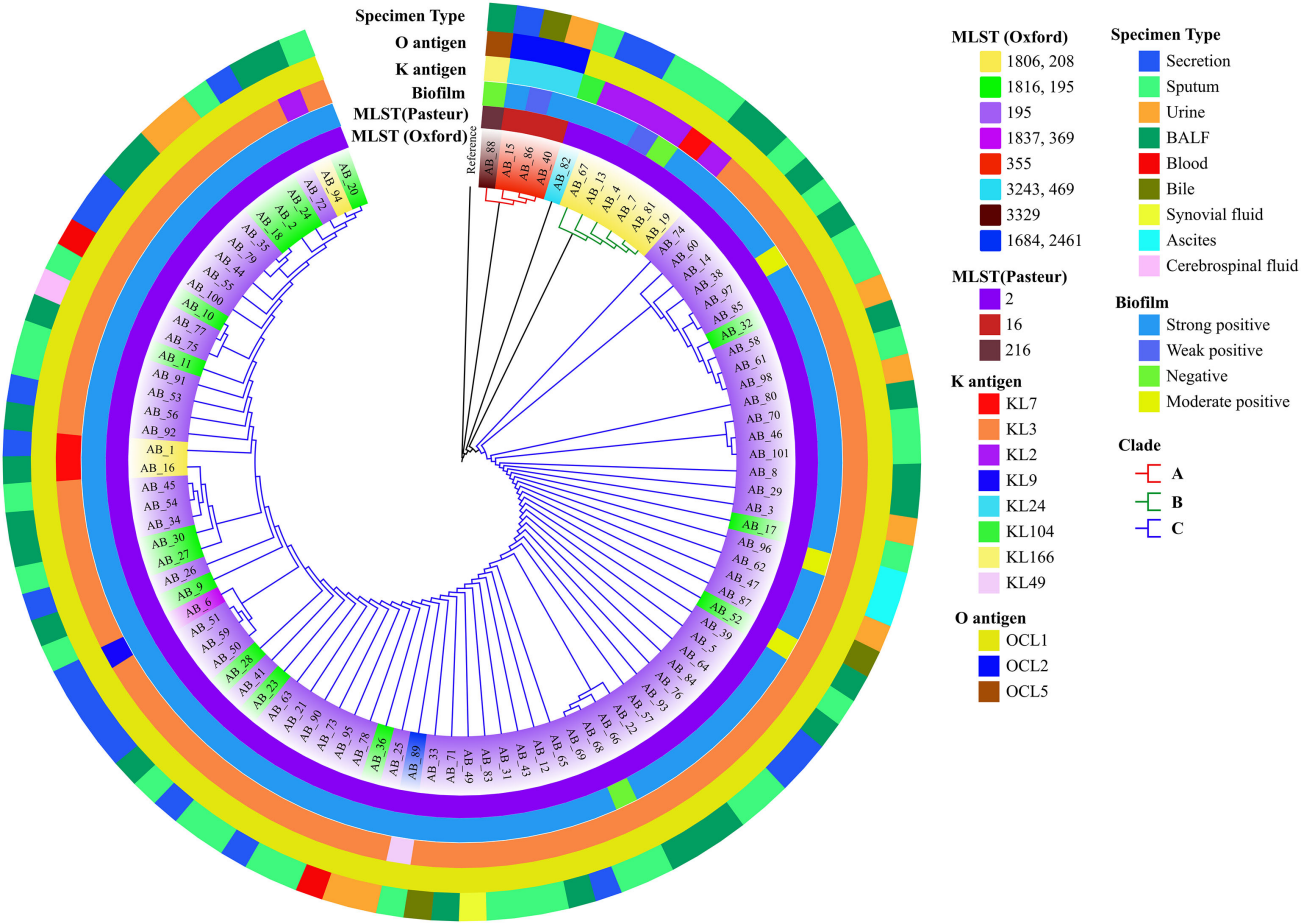
Phylogenetic tree of the 97 CRAB isolates based on core−genome single−nucleotide polymorphisms (SNPs). The phylogenetic tree clustered the isolates into three major Clades (A, B, and C). Clade C comprised the largest number of isolates, predominantly belonging to ST2 (Pasteur scheme), with ST195, 1816 (Oxford scheme) also present; KL3 and OCL1 were also mainly distributed within Clade C. Strong biofilm−forming isolates (91.75%) were distributed across all clades. From the innermost to the outermost rings: Innermost ring: Clades (Clade A in red, Clade B in green, Clade C in blue). Second ring: isolate identifiers and Oxford MLST types. Third ring: Pasteur MLST types. Fourth ring: biofilm formation categories. Fifth ring: KL types. Sixth ring: OCL types. Outermost ring: specimen types. **Reference:** *Acinetobacter baumannii* ACICU (GenBank accession no. GCF_000018445.1).

Under the Pasteur scheme (**Figure 2**), three sequence types (ST) were identified among the 97 CRAB isolates. ST2 was overwhelmingly predominant (95.88%), followed by ST16 (3.09%) and ST216 (1.03%). The Oxford scheme, which provides higher resolution, revealed eight ST: ST195 was the most common (68.04%), followed by ST1816/ST195 (15.46%) and ST1806/ST208 (9.28%); the remaining 7.22% comprised other types.

A SNP-based phylogenetic tree divided the 97 isolates into three main clades (Clades A, B and C; see section 3.6). Under the Pasteur scheme, Clade A was dominated by ST16, while Clades B and C both consisted of ST2 isolates, indicating that the CRAB population in our hospital is dominated by the ST2 clone (Clades B and C) and that the ST16 clone (Clade A) represents a relatively independent lineage. According to the Oxford scheme, Clade A mainly corresponded to ST335, Clade B to ST1806/ST208, and Clade C mainly to ST195 and ST1816/ST195.

### 3.4 Capsular types, virulence gene profiles and biofilm formation

Capsular typing identified eight different KL types. KL3 was the most frequent (83.51%), followed by KL2 (6.19%), KL24 and KL7 (3.09% each). Three OCL types were detected: OCL1 was predominant (95.88%); OCL2 was found in three isolates (3.09%, all of which were KL24); and one isolate (1.03%) was OCL5. See **Figure 2** for details.

**Figure 3:**
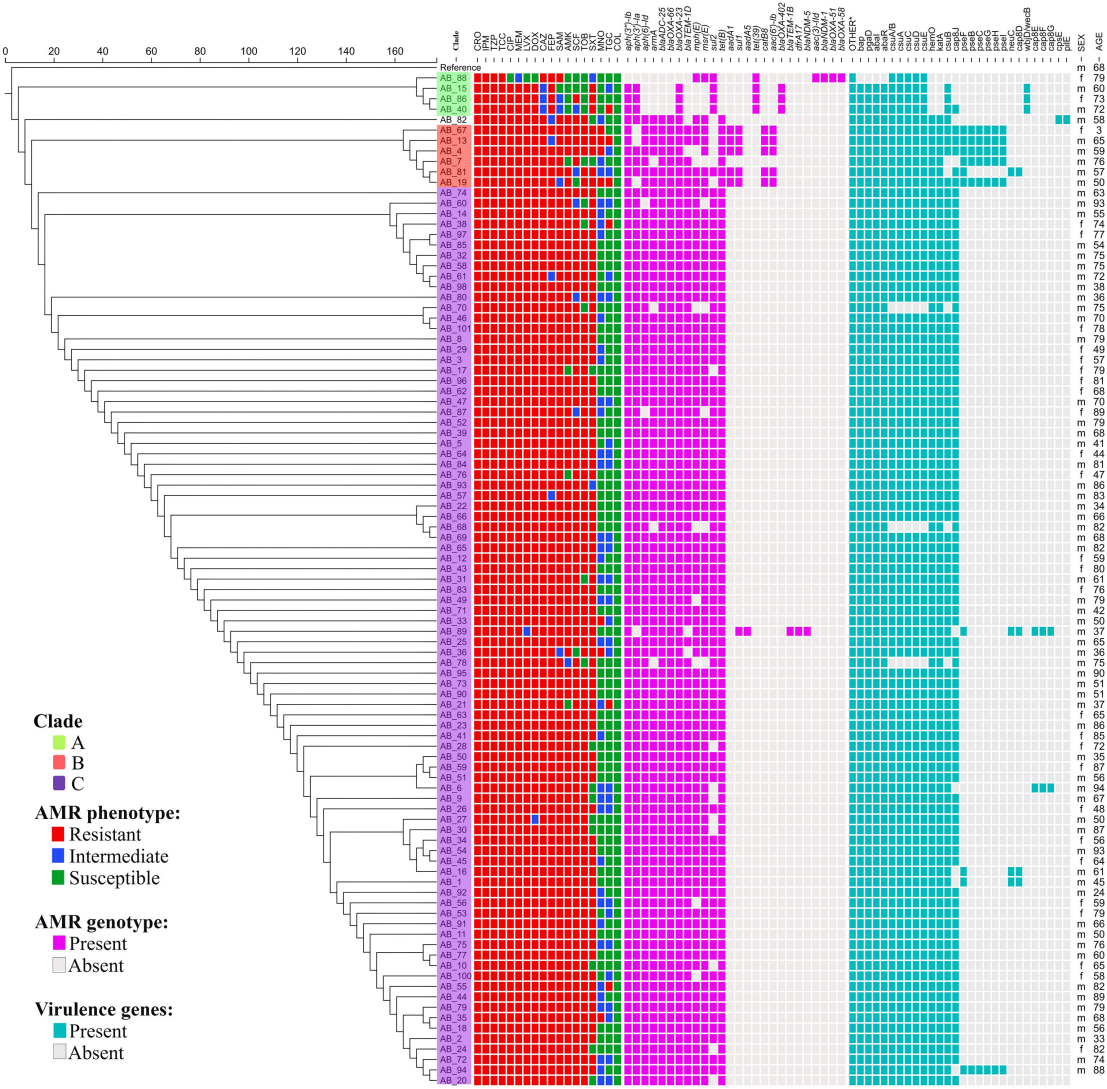
Phylogenetic tree integrated with heatmaps of antimicrobial resistance (AMR) phenotypes, AMR genotype and virulence genes of the 97 CRAB isolates. Clade A carried fewer resistance genes and lacked *bla*OXA−66, *aph(6)−Id*, *armA*, *bla*ADC−25, *bla*TEM−1D, *mph(E)*, *msr(E)*, and *tet(B)*, which were commonly present in Clades B and C. Compared with Clade C, Clade B additionally carried *catB8*, *aadA1*, and *aac(6′)−Ib*, and harboured *sul1* in place of *sul2*. These genotypic differences were consistent with the phenotypic resistance profiles: Clade A isolates were susceptible to amikacin, tobramycin, and minocycline, whereas Clades B and C were resistant. From left to right: the leftmost part shows the core-genome phylogenetic tree. The adjacent heatmap represents AMR phenotypes: resistant (red), intermediate (blue) and susceptible (green). The next heatmap shows AMR genotype: Present (purple)/Absent (white). The following heatmap indicates virulence genes: Present (cyan)/Absent (white). The rightmost columns display patient sex and age. Reference: *Acinetobacter baumannii* ACICU (GenBank accession no. GCF_000018445.1). Note: “Other” in the virulence gene heatmap denotes genes that were present in all isolates, including *ompA*, *adeFGH*, *pgaABC*, *plcCD*, *cap8P*, *lpxABCDLM*, *barAB*, *basABCDFGHIJ*, *bauABCDEF*, *bfmRS* and *pbpG*.

A total of 73 virulence genes were detected, with each isolate carrying 51–65 (mean 59). All isolates harboured key virulence genes such as *ompA*, *adeFGH*, *pgaABC*, *plcCD*, *cap8P*, *lpxABCDLM*, *barAB*, *basABCDFGHIJ*, *bauABCDEF*, *bfmRS* and *pbpG*. In addition, the carriage rates of biofilm-associated genes (*bap*, *csuABCDE*, *pgaD*), quorum-sensing genes (*abaI/R*), the iron-uptake gene (*hemO*) and the stress-adaptation gene (*katA*) all exceeded 90%. Differences in virulence gene profiles were observed among clades: Clade A lacked *hemO*, *katA* and *cap8J* but carried *wbjD/wecB*, whereas Clade B carried *pseBCFGHI*. Detailed information is shown in **Figure 3**.

Quantitative crystal violet assay (**Figure 2**) showed that 91.75% of isolates were strong biofilm formers, 3.09% moderate, 2.06% weak, and only 3.09% were non-producers. Thus, 94.84% of CRAB isolates displayed moderate-to-strong biofilm formation, consistent with the high prevalence of biofilm-associated virulence genes (*bap*, *csuABCDE*, *pgaD*, etc.).

### 3.5 Resistance gene profiles

A total of 26 acquired resistance genes were detected across the 97 isolates (**Figure 3**), with each isolate carrying 6–16 genes (mean 12). All isolates carried intrinsic *blaOXA-51-like* genes, predominantly *blaOXA-66* (95.88%). The most common carbapenemase gene was *blaOXA-23* (98.97%). Two isolates carried metallo-β-lactamase (MBL) genes: one co-carried *blaNDM-1* and *blaOXA-58*, and the other co-carried *blaNDM-5* and *blaOXA-23*. *IMP*, *VIM* or *KPC* were not detected.

Carriage rates of other resistance genes were as follows: β-lactamase genes *blaADC-25* (95.88%) and *blaTEM-1D* (91.75%); aminoglycoside-modifying enzyme genes *aph(3″)-Ib* (98.97%), *aph(3′)-Ia* (94.85%), *aph(6)-Id* (93.81%) and *armA* (91.75%); tetracycline efflux pump gene *tet(B)* (95.88%); macrolide resistance genes *mph(E)* (88.66%) and *msr(E)* (90.72%); and sulphonamide resistance gene *sul2* (84.5%). No *mcr*-type colistin resistance genes were found.

When examined in the context of the phylogenetic tree, marked differences in resistance gene profiles were observed among clades. Clade A (ST16) lacked *blaOXA-66*, *aph(6)-Id*, *armA*, *blaADC-25*, *blaTEM-1D*, *mph(E)*, *msr(E)* and *tet(B)*, but carried *blaOXA-402* (another *blaOXA-51-like* variant) and *tet(39)*, which were absent from Clades B and C. Clade B carried additional genes *catB8*, *aadA1* and *aac(6′)-Ib*, and had *sul1* instead of *sul2*. These genetic differences correlated perfectly with the resistance phenotypes: Clade A was susceptible to amikacin, tobramycin and minocycline, whereas Clades B and C were resistant. Details are shown in **Figure 3**.

### 3.6 Phylogeny, clonal clades and phenotypic associations

Pairwise SNP distances among the 97 isolates ranged from 0 to 103. Isolates with ≤15 SNPs were found in different departments throughout the hospital, suggesting possible cross-departmental transmission. The phylogenetic tree divided the isolates into three major clades (A, B and C), comprising 4.12%, 6.19% and 88.66% of the isolates, respectively. The vast majority of isolates were concentrated in Clade C.

**Figure 4:**
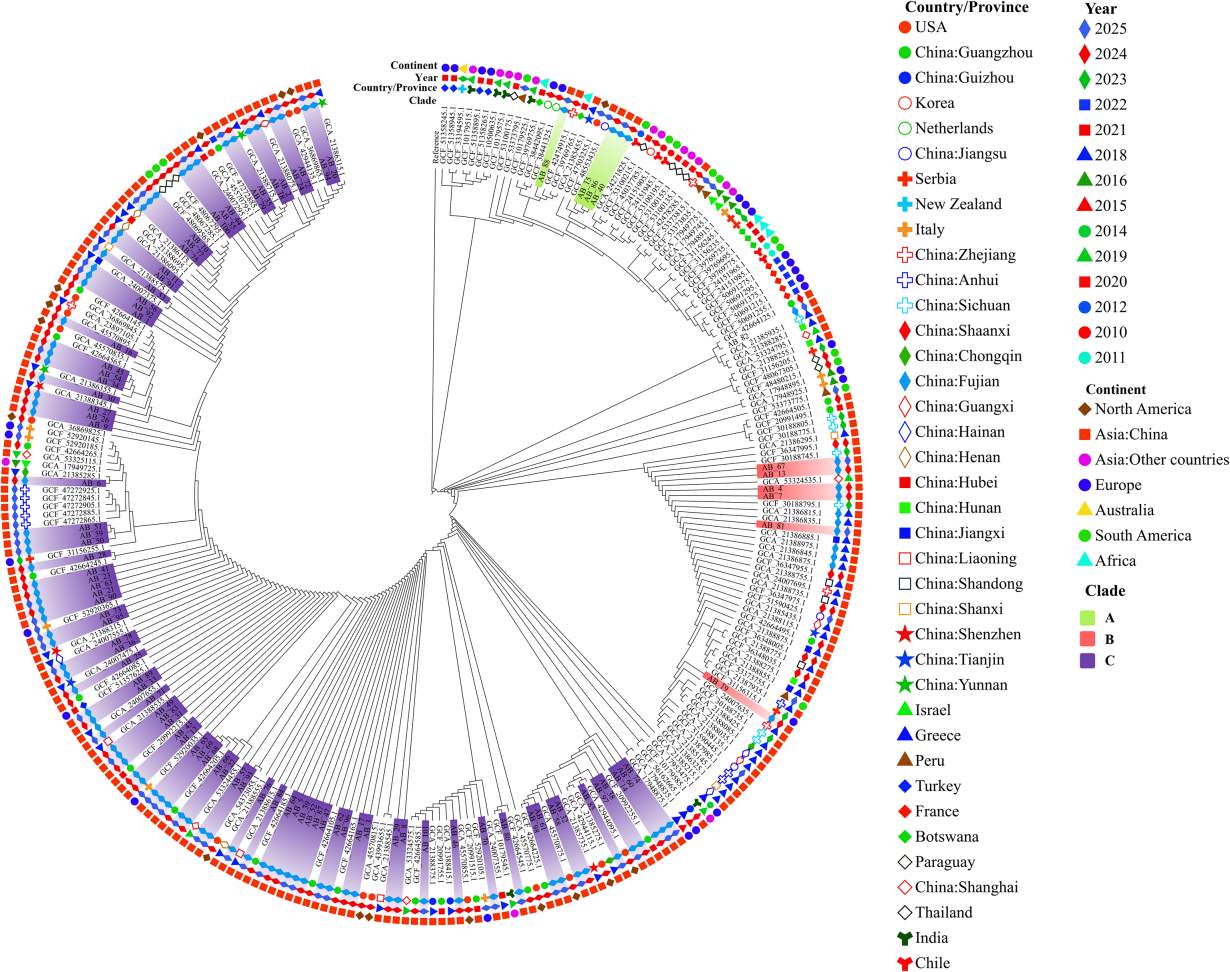
Phylogenetic tree of 284 global CRAB isolates based on core−genome single−nucleotide polymorphisms (SNPs). Clade A (ST16/KL24/OCL2) was distributed across all six continents. Clade B (ST2/KL2/OCL1) and Clade C (ST2/KL3/OCL1) were predominantly circulating in China. Clade B emerged as an outbreak cluster after 2018. Clade C entered its outbreak phase in 2018 and became the dominant clone in our hospital and across southeastern China during 2024–2025, exhibiting a trend of gradually replacing Clade B. From the innermost to the outermost rings: First ring: distribution of the 97 isolates from this study (highlighted in a distinct colour) among all global isolates. Second ring: country of origin (for non−Chinese isolates) or province of China. Third ring: year of isolation. Outermost ring: continent of origin. Reference: *Acinetobacter baumannii* ACICU (GenBank accession no. GCF_000018445.1).

Global analysis (**Figure 4**) showed that Clade A (ST16/KL24/OCL2) was distributed across all six continents and spanned a long time period (2010–2025). In contrast, Clades B (ST2/KL2/OCL1) and C (ST2/KL3/OCL1) were mainly confined to China, but their temporal dynamics differed. Clade B emerged as an outbreak after 2018, gradually declined but continued to be detected until 2025. Clade C had a more complex trajectory: sporadic isolates appeared as early as 2014, suggesting early colonisation; it entered an outbreak phase after 2018 and became the dominant clone in our hospital and in Southeast China during 2024–2025, progressively replacing Clade B.

## 4 Discussion

Orthopaedic patients often receive implants (e.g., for fracture fixation or joint replacement) and experience prolonged hospital stays, frequent antibiotic use and many post-operative invasive procedures, all of which facilitate CRAB colonisation and infection (Asokan S. et al., 2026). In this study, 17.5% of isolates came from orthopaedic wards and 18.6% from wound secretions, indicating that CRAB is an important pathogen in post-operative orthopaedic wound and implant-related infections. Although these proportions are lower than those in ICU, orthopaedic patients have a high rate of implant exposure and a long wound healing period; once CRAB infection occurs, it often leads to difficult treatment, extended hospitalisation and even the need for a second operation (Vasso et al., 2022). Therefore, understanding the epidemiological features of CRAB in orthopaedic specialty hospitals is of great significance for guiding clinical infection prevention and control.

ST2 belongs to global clone 2 (GC2) and has been shown to be the most prevalent CRAB lineage in Chinese general hospitals. Liu et al. reported that 99.2% of CRAB isolates from ICUs in 77 nationwide hospitals were ST2 (Liu et al., 2022), and Wang et al. found that 84.13% of CRAB isolates from several hospitals in Shanghai were ST2 (Wang et al., 2025). Globally, ST2 is also a dominant CRAB clone (Wang M. et al., 2024). In the present study, ST2 was overwhelmingly predominant (95.88%), consistent with the trends described above, indicating that this clone also dominates in orthopaedic specialty hospitals. This study extends the known prevalence of ST2 to the orthopaedic specialty setting for the first time. Notably, the proportion of ST2 in our study (95.88%) was even higher than that reported in most general hospital ICUs (Chen et al., 2025; Wang et al., 2025; Chukamnerd et al., 2026), possibly due to the long-term use of broad-spectrum antibiotics in orthopaedic wards and the sustained selective pressure of implant surfaces.

The vast majority of isolates (98.97%) carried *blaOXA-23*, the main carbapenem resistance determinant (Wong et al., 2019; Wei et al., 2024). This gene is highly prevalent in Chinese CRAB populations, with consistently high frequencies across different regions and hospital types (Pu et al., 2019; Zhang et al., 2021; Gu et al., 2022; Liu L. et al., 2024). Notably, two isolates in this study co-carried metallo-β-lactamase (MBL) genes (*blaNDM-1* and *blaNDM-5*, respectively). Although still uncommon, the coexistence of *blaOXA-23* and MBL genes has been increasingly reported (Liu H. et al., 2024; Xu et al., 2024). MBLs hydrolyse almost all β-lactams and are not inhibited by commonly used β-lactamase inhibitors (tazobactam, sulbactam, avibactam) (Mojica et al., 2016). The novel inhibitor combination sulbactam-durlobactam is active against *blaOXA-23* producing strains but not against MBL producers (McLeod et al., 2020). Such co-carrying isolates pose a major challenge to clinical treatment. Therefore, rapid identification of MBL-positive CRAB is critical for guiding precise therapy and for implementing timely measures to prevent further spread. Although no colistin-resistant isolate was detected in this study and no plasmid-borne *mcr* gene was found, colistin-resistant CRAB strains carrying *PmrA* or *PmrB* variants have been increasingly reported both in China and internationally (Lunha et al., 2020; Acharya et al., 2025), indicating that colistin resistance can still emerge via chromosomal mutations. Hence, these “last-line” agents should still be used with caution in clinical practice. From an infection control policy perspective, orthopaedic institutions should establish a local surveillance system for CRAB resistance genes and designate CRAB as a priority target pathogen in perioperative management for implant surgery. When empirical therapy with limited agents such as polymyxins, tigecycline, or sulbactam-durlobactam is considered, susceptibility testing and resistance gene detection (e.g., MBL, *mcr, PmrB*, or *PmrA*) should be performed concurrently. Subsequent therapeutic regimens should be adjusted promptly based on susceptibility results and resistance genotypes, with preference given to combination therapy to reduce the risk of treatment failure, while strictly limiting the duration of therapy to delay the selective expansion of resistant mutants.

All isolates in this study carried a complete set of biofilm-associated genes (*csuABCDE*, *bap*, *pgaABCD*, *ompA*), and 91.75% of the isolates were strong biofilm formers – a rate considerably higher than those typically reported in general hospitals (usually 30-50%) (Liu Z. et al., 2026). The specificity of the orthopaedic hospital may explain this difference. Orthopaedic implants (joint prostheses, fracture fixation devices) offer abiotic surfaces for biofilm formation. Under repeated antibiotic exposure and host immune pressure, CRAB strains that persistently colonise implants may be positively selected for stronger biofilm-forming ability. Once formed, biofilms protect bacteria from antibiotics and host immune clearance, leading to persistent infection (Asokan S. P. R. et al., 2026; Liu M. et al., 2026). Therefore, for high−risk patients undergoing implant surgery, biofilm prevention strategies should be reinforced during the perioperative period, and the biofilm−forming capacity of CRAB should be incorporated into infection risk assessment. In cases of CRAB infection complicated by the presence of an implant, clinical management should not rely excessively on prolonged antibiotic courses alone. Instead, priority should be given to early debridement, adequate drainage, and implant removal when necessary as source control measures, rather than reserving these interventions as salvage options after antibiotic failure. Furthermore, in this study, KL3 accounted for 83.51% of the isolates and was almost exclusively associated with the ST2 clone (Clade C). The KL type is an important virulence determinant of *A. baumannii*, affecting resistance, biofilm formation and immune evasion (Geisinger and Isberg, 2015; Singh et al., 2018). KL2 has long been considered the most prevalent capsular type among CRAB (Zhang et al., 2026), but in this study KL3 predominated. This discrepancy may reflect regional differences in clonal distribution, antibiotic selection pressure, immune evasion properties, or enhanced adaptability of this capsular type to implant surfaces in orthopaedic patients (Way et al., 2024; Deng et al., 2025; Jiang et al., 2025). However, there is currently no evidence to suggest that KL3 is superior to KL2 in terms of biofilm formation ability, colonization duration, or treatment failure rate. Further verification is needed by combining longitudinal follow-up data with functional experiments (such as capsule gene knockout and complement killing experiments).

The global phylogenetic tree showed that the circulating strains in our hospital comprised three main clades. Clade A (ST16/KL24) was distributed across all continents and spanned a long time period, but accounted for only a small proportion in our hospital. Clade B (ST2/KL2) circulated mainly in China after 2018 and has gradually been replaced by Clade C (ST2/KL3) in recent years. The latter became the dominant clone in 2024-2025, and a capsular switch from KL2 to KL3 was observed. It has been reported that capsular switching occurs rather frequently during the dissemination of the ST2 clone and may be associated with its clonal spread (Diep et al., 2023). As the most recently circulating clone, Clade C possesses a stronger biofilm-forming ability, possibly reflecting an evolutionary shift from “accumulation of resistance genes” towards “optimisation of virulence/colonisation capacity” – a shift that would confer an adaptive advantage under the selective pressure of orthopaedic implant surfaces. SNP analysis also showed that CRAB isolates in this study were distributed across different departments throughout the hospital, suggesting possible cross-departmental transmission. However, because we did not conduct patient movement analysis, healthcare worker screening, or environmental investigation, we cannot confirm the actual transmission routes. Based on previous reports, we speculate that potential vehicles include healthcare workers’ hands, shared medical equipment, and patient transfers between wards (E et al., 2026). Strengthened isolation measures between departments are recommended, and routine surveillance should include KL typing and assessment of biofilm−forming phenotype.

This study has several limitations. First, it is a single-centre retrospective study with a relatively limited sample size; further data accumulation is needed to confirm the generalisability of the findings. Second, the absence of environmental sampling and screening of healthcare workers prevented complete reconstruction of transmission pathways. Third, due to the lack of prospective follow−up and uniformly applied standardized infection definitions for all patients, some uncertainty may remain in a subset of cases, and it is possible that infection versus colonization could not be definitively distinguished for individual isolates. Fourth, the biofilm formation assay was performed under static *in vitro* conditions. This approach does not distinguish viable from dead bacteria within the biofilm, nor does it reveal its three-dimensional structural features, nor can it fully reflect the structure of the biofilm under physiological conditions. Future research should combine multiple complementary methods, including confocal laser scanning microscopy with live/dead staining, scanning electron microscopy, and flow cytometry systems, to further verify the results. Fifth, transcriptomic profiling, functional gene validation, and *in vivo* infection model experiments were not performed in the present study. Therefore, the expression levels of resistance or virulence genes, the causal relationship between genotype and phenotype, and the pathogenic potential or biofilm-forming capacity under physiological conditions remain to be further elucidated in future investigations.

## 5 Conclusions

CRAB in this orthopaedic specialty hospital is dominated by the ST2/KL3 clone, which carries multiple resistance and virulence genes, exhibits a remarkably strong biofilm-forming ability, and shows a capsular switch trend from KL2 to KL3. Enhanced molecular surveillance of this dominant clone and increased attention to anti_-_biofilm strategies for orthopaedic implant-related infections are strongly recommended.

## Data Availability

The datasets presented in this study can be found in online repositories. The names of the repository/repositories and accession number(s) can be found below: https://www.ncbi.nlm.nih.gov/, PRJNA1474437.
