# Supplementary material for: Genomic characterization of carbapenem-resistant Acinetobacter baumannii from a specialized orthopaedic hospital in Southeast China, with phenotypic analysis of biofilm formation
Source: Front Cell Infect Microbiol. 2026 Jul 24;16:1904047. doi: 10.3389/fcimb.2026.1904047 (PMC13447295; doi:10.3389/fcimb.2026.1904047)
Supplement: Supplementary file 1 [file Table1.docx]

### **Table S1 Resistance pattern of 97 clinical CRAB isolates**

| **Resistance pattern** | **N (%)** |
| --- | --- |
| SAM＋CRO＋AMK＋IPM＋LVX＋MEM＋SXT＋TOB＋FEP＋CAZ＋TZP＋TCC＋DOX＋SCF＋CIP | 60(61.86) |
| SAM＋CRO＋AMK＋IPM＋LVX＋MEM＋TOB＋FEP＋CAZ＋TZP＋TCC＋DOX＋SCF＋CIP | 8(8.25) |
| SAM＋CRO＋AMK＋IPM＋LVX＋MEM＋SXT＋TOB＋MNO＋FEP＋CAZ＋TZP＋TCC＋DOX＋SCF＋CIP | 4(4.12) |
| SAM＋CRO＋AMK＋IPM＋LVX＋MEM＋SXT＋TOB＋FEP＋CAZ＋TZP＋TCC＋DOX＋CIP | 3(3.09) |
| SAM＋CRO＋AMK＋IPM＋LVX＋MEM＋SXT＋FEP＋CAZ＋TZP＋TCC＋DOX＋SCF＋CIP | 2(2.06) |
| SAM＋CRO＋AMK＋IPM＋LVX＋MEM＋SXT＋TOB＋CAZ＋TZP＋TCC＋DOX＋SCF＋CIP | 2(2.06) |
| CRO＋AMK＋IPM＋LVX＋MEM＋SXT＋TOB＋MNO＋FEP＋CAZ＋TZP＋TCC＋DOX＋CIP | 1(1.03) |
| CRO＋AMK＋IPM＋LVX＋MEM＋SXT＋TOB＋MNO＋FEP＋CAZ＋TZP＋TCC＋DOX＋CIP＋TGC | 1(1.03) |
| CRO＋IPM＋LVX＋MEM＋SXT＋FEP＋TZP＋TCC＋DOX＋CIP | 1(1.03) |
| CRO＋IPM＋LVX＋MEM＋SXT＋FEP＋TZP＋TCC＋DOX＋CIP＋TGC | 1(1.03) |
| CRO＋IPM＋LVX＋MEM＋SXT＋FEP＋TZP＋TCC＋DOX＋SCF＋CIP | 1(1.03) |
| SAM＋CRO＋AMK＋IPM＋LVX＋MEM＋SXT＋FEP＋CAZ＋TZP＋TCC＋DOX＋CIP | 1(1.03) |
| SAM＋CRO＋AMK＋IPM＋LVX＋MEM＋SXT＋FEP＋CAZ＋TZP＋TCC＋DOX＋SCF＋CIP＋TGC | 1(1.03) |
| SAM＋CRO＋AMK＋IPM＋LVX＋MEM＋SXT＋TOB＋FEP＋CAZ＋TZP＋TCC＋DOX＋SCF＋CIP＋TGC | 1(1.03) |
| SAM＋CRO＋AMK＋IPM＋LVX＋MEM＋SXT＋TOB＋MNO＋CAZ＋TZP＋TCC＋DOX＋SCF＋CIP＋TGC | 1(1.03) |
| SAM＋CRO＋AMK＋IPM＋LVX＋MEM＋TOB＋CAZ＋TZP＋TCC＋DOX＋SCF＋CIP | 1(1.03) |
| SAM＋CRO＋AMK＋IPM＋LVX＋MEM＋TOB＋FEP＋CAZ＋TZP＋TCC＋SCF＋CIP | 1(1.03) |
| SAM＋CRO＋AMK＋IPM＋MEM＋SXT＋TOB＋FEP＋CAZ＋TZP＋TCC＋DOX＋SCF＋CIP | 1(1.03) |
| SAM＋CRO＋IPM＋FEP＋CAZ＋TZP＋TCC | 1(1.03) |
| SAM＋CRO＋IPM＋LVX＋MEM＋FEP＋CAZ＋TZP＋TCC＋DOX＋SCF＋CIP | 1(1.03) |
| SAM＋CRO＋IPM＋LVX＋MEM＋SXT＋FEP＋CAZ＋TZP＋TCC＋DOX＋SCF＋CIP | 1(1.03) |
| SAM＋CRO＋IPM＋LVX＋MEM＋SXT＋TOB＋FEP＋CAZ＋TZP＋TCC＋DOX＋SCF＋CIP | 1(1.03) |
| SAM＋CRO＋IPM＋LVX＋MEM＋SXT＋TOB＋FEP＋CAZ＋TZP＋TCC＋DOX＋SCF＋CIP＋TGC | 1(1.03) |
| SAM＋CRO＋IPM＋LVX＋MEM＋TOB＋FEP＋CAZ＋TZP＋TCC＋DOX＋SCF＋CIP | 1(1.03) |

**Note:** SAM, ampicillin‑sulbactam; CRO, ceftriaxone; AMK, amikacin; IPM, imipenem; LVX, levofloxacin; MEM, meropenem; SXT, trimethoprim‑sulfamethoxazole; TOB, tobramycin; FEP, cefepime; CAZ, ceftazidime; TZP, piperacillin‑tazobactam; TCC, ticarcillin‑clavulanate; DOX, doxycycline; SCF, cefoperazone‑sulbactam; CIP, ciprofloxacin; MNO, minocycline; TGC, tigecycline.
